# Early clinical outcomes of ınferior turbinate radiofrequency and lateralization combined with septoplasty

**DOI:** 10.1016/j.bjorl.2020.07.004

**Published:** 2020-08-19

**Authors:** Dursun Mehmet Mehel, Tuğba Yemiş, Mehmet Çelebi, Erkan Can, Doğukan Özdemir, Asude Ünal, Abdulkadir Özgür

**Affiliations:** a University of Health Sciences Turkey, Samsun Health Practices and Research Center, Department of Otorhinolaryngology, Samsun, Turkey; b Gümüşhane State Hospital, Department of Otorhinolaryngology, Gümüşhane, Turkey

**Keywords:** Septoplasty, Inferior turbinate, Radiofrequency, Lateralization, NOSE

## Abstract

**Introduction:**

Mechanical obstruction is the most common form of nasal obstruction. Among the types of mechanical obstructions, septum deviation and inferior turbinate hypertrophy are the most prevalent.

**Objective:**

This study evaluated the early clinical outcomes of inferior turbinate radiofrequency and inferior turbinate lateralization combined with septoplasty in the treatment of nasal obstruction symptoms.

**Methods:**

The research retrospectively evaluated data from 33 patients (24 male, nine female) undergoing septoplasty and inferior turbinate radiofrequency (RF group) and 32 patients (24 male, eight female) treated with septoplasty and inferior turbinate lateralization (LAT group), who were admitted, with complaints of nasal obstruction, to the University of Health Sciences, Department of Otorhinolaryngology, between January 1, 2017 and January 1, 2018. The patients’ preoperative and 6-month postoperative symptoms were evaluated via the Nasal Obstruction Symptom Evaluation, the NOSE scale.

**Results:**

The mean preoperative NOSE scores were 10.3 ± 4.2 in the RF group and 10.9 ± 4.9 in the LAT group, and the mean six-month postoperative scores were 1.09 ± 1.3 in the RF group and 1.2 ± 1.3 in the LAT group. There was no significant difference in NOSE scores between the two groups (*p* > 0.05).

**Conclusion:**

The data obtained in this study show that both methods result in similar outcomes in terms of relieving nasal obstruction symptoms in patients requiring inferior turbinate intervention. Therefore, the researchers believe that, in each case, the intervention method should be selected at the discretion of the patient and surgeon(s).

## Introduction

The most common form of nasal obstruction is mechanical obstruction. Among the types of mechanical obstructions, septum deviation and inferior turbinate hypertrophy are the most prevalent.^1^ Medical treatments are primarily applied to the inferior turbinate hypertrophies that develop due to vasomotor or allergic rhinitis.^2^ Turbinectomy, submucosal resection, thermal or chemical coagulation, vidian neurectomy, the lateralization (out-fracture) technique, and laser vaporization have been described for use in cases in which medical treatment has failed. Turbinate surgery aims to reduce the volume of tissue while preserving mucociliary function; however, most methods do not achieve this goal.^3^, ^4^ Inferior turbinate radiofrequency and inferior turbinate lateralization are frequently preferred because of their successful outcomes and low rates of complications (e.g., bleeding and crusting).^5^ Although reliable objective criteria and methods have been developed for evaluating nasal function, there is still little correlation between the findings of objective examinations and patients’ subjective findings.^6^ The Nasal Obstruction Symptom Evaluation (NOSE) scale was developed to assess nasal obstruction and disease-specific outcomes. This validated method has also been used to evaluate the treatment of nasal obstruction accompanied by turbinate surgery.^7^ The present study, therefore, used the NOSE scale to evaluate the early clinical outcomes of inferior turbinate radiofrequency and inferior turbinate lateralization methods, combined with septoplasty, among patients in the researchers’ clinic.

## Methods

Patients were included in this study if they were admitted to the Otorhinolaryngology Clinic of the researchers’ hospital (a tertiary healthcare institution) for nasal obstruction and underwent septoplasty with inferior turbinate lateralization (LAT group) or radiofrequency (RF group) between January 1, 2017 and January 1, 2018. Ethics committee approval was obtained from the researchers’ institution (no. 35-2018/2-12), and the study was performed in accordance with the Declaration of Helsinki. Written informed consent was obtained from all participants. Patients with nasal polyps, chronic sinusitis, nasal valve insufficiency, septal perforation and septal synechia, or obstruction of the adenoid and concha bullosa were excluded from the research, as were those who were treated only with inferior turbinate radiofrequency or inferior turbinate lateralization (i.e., without septoplasty).

### Septoplasty

All surgical procedures were performed under general anesthesia. After the vasoconstrictor (1% lidocaine with 1:100.000 epinephrine) was injected into the septal mucosa, the caudal septum was made visible with a small nasal speculum, and a hemitransfixion incision was made with a #15 scalpel. At the submucopericondrial level, the septal cartilage was made visible by elevation of the mucoperichondrium with a Cottle elevator. Curved parts of the septal cartilage were excised, leaving sufficient supportive cartilage in the dorsal and caudal septum. The mucosa was sutured with a 4/0 round needle soluble suture to prevent septal hematoma.

### Radiofrequency

The Celonlab ENT (Olympus America) system was used for inferior turbinate radiofrequency treatment. Physiological serum (0.9% NaCl) was injected into the anterior section of both inferior turbinates with the help of a 22G × 32 mm dental syringe at a dose of 2–4 mL per application. The radiofrequency applicator was inserted through the anterior tip of the turbinate and was submucosally advanced to the turbinate tail without making contact with the turbinate bone. 10 W of energy was then applied. This process was repeated at three different points.

### Lateralization

For inferior turbinate lateralization treatment, Cottle elevators were employed to first medialize and then lateralize the turbinate to form a greenstick fracture. At the same time, care was taken not to damage the mucosa. Merocel nasal tampons were used for hemostasis after surgical procedures. All patients received amoxicillin–clavulanic acid (1 g/day) and paracetamol (500 mg twice daily) as antibiotic treatment for seven days.

Patients’ preoperative and six month postoperative symptoms were evaluated via the NOSE scale, which was validated in Turkish.^8^ The scale's five questions were posed to the patients before and after the procedures and were scored on a four-point system according to the level of complaint (severe = 4; moderate = 3, mild = 2, minimal = 1, absent = 0).

## Results

**Figure 1:**
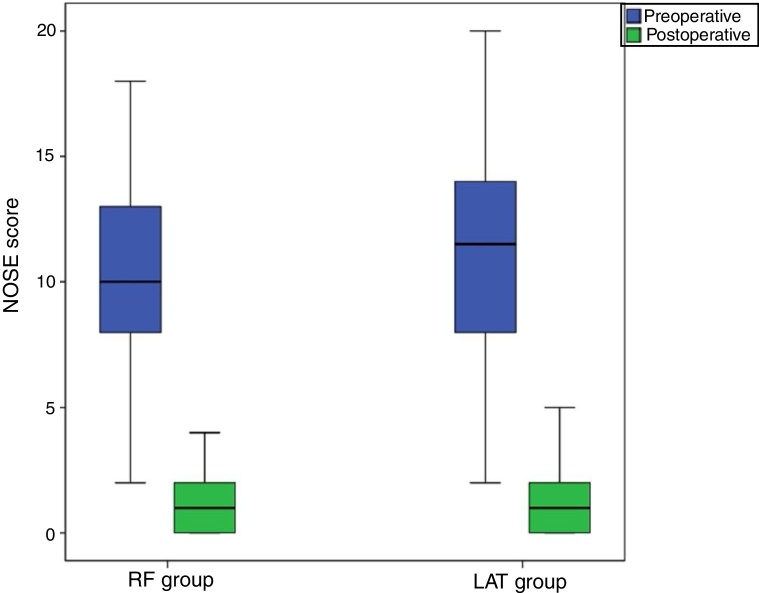
Preoperative and postoperative NOSE scores.

In this study, 33 patients (24 males, nine female) were included in the RF Group, with a mean age of 26.7 ± 8.7 years. 32 patients (24 males, 8 female) were included in the LAT Group, with a mean age of 28.7 ± 11.5 years. There was no significant difference between the groups in terms of age or gender (*p* > 0.05). Mean preoperative NOSE scores were 10.3 ± 4.2 in the RF Group and 10.9 ± 4.9 in the LAT Group. Mean six-month postoperative NOSE scores were 1.09 ± 1.3 in the RF Group and 1.2 ± 1.3 in the LAT Group (Fig. 1). The NOSE score decreased postoperatively by 9.2 in the RF Group and by 9.8 in the LAT Group. There was no significant difference in postoperative NOSE scores between the RF and LAT Groups (*p* > 0.05). Postoperative intranasal crusting occurred in the RF Group during the early period. No complications however were observed at the late postoperative period in either the RF or the LAT Group.

## Discussion

Turbinate reduction surgery aims to reduce the resistance of air flow in the nose, reduce nasal discharge, and improve the patient's quality of life by diminishing headaches, snoring, and sleep apnea. There is no excellent technique for turbinate surgery, but the procedure's main objective should be to protect the mucosal surface while reducing the volume of the submucosal tissue. However, most extant techniques for this process do not provide the desired results to preserve nasal function. The variety of applicable surgical techniques is indicative of the lack of consensus of an optimal technique.^5^ In the literature, the Visual Analog Scale (VAS), rhinomanometry, and acoustic rhinometry are commonly employed, both pre- and postoperatively, to evaluate the efficacy of turbinate surgery.^9^ However, nasal obstruction symptoms are examined only in the VAS–not by rhinomanometry or acoustic rhinometry. The NOSE scale, implemented in this study, evaluates the symptoms of nasal fullness, nasal congestion, difficulty breathing through the nose, difficulty breathing through the nose during sleep, and difficulty breathing through the nose during exercise or movement. In the present case, the results of two different interventions applied to the lower concha, in conjunction with septoplasty, were examined with the NOSE scale, and the resulting data has shown that radiofrequency or lateralization procedures, applied to the lower turbinate, decreased symptom scores in both the RF and LAT Groups; however, there was no significant difference between the groups.

In the radiofrequency procedure, energy induces ion stimulation in the tissue, increases local temperature, and causes a thermal lesion, which is intended to occur in the deep mucosa without damaging the surface.^6^ Thus, the process may lead to submucosal fibrosis through the coagulation of venous sinusoids within the turbinate.^10^ Şapçi et al. compared the effects of laser treatment, partial turbinectomy, and radiofrequency surgery on mucociliary transport. They reported that radiofrequency surgery either did not cause nasal mucosal damage or only caused minimal damage.^11^ In radiofrequency studies, Utley et al. and Li et al. reported success rates of 100% and 86.4%, respectively.^2^, ^12^ Seeger et al. found that 68% of their sampled patients postoperatively formed crusts lasting for 5 days, and, in one case, the crust remained for 6 weeks.^13^ However, Rhee et al. and Coste et al. asserted that they did not observe any crusting or dryness.^8^, ^14^ In the present study, mild crusting occurred in 20 patients. However, these crusts resolved completely in the first postoperative month.

Lateralization of the turbinate reduces tissue via a greenstick fracture. The healing process secondarily induces fibrosis by wound contraction, which leads to a reduction in tissue volume. Büyüklü and Zhang stated that conchal lateralization applied to inferior turbinate hypertrophy effectively expands nasal passages.^15^, ^16^ Turbinoplasty causes a more pronounced widening in the passage due to decreased bone mass. However, the lateralization and radiofrequency methods are applicable to moderate- and low-grade turbinate hypertrophies since the complication risk is low.^17^ In the present study, no major complications were observed in either group.

This research used only symptom scores as clinical evaluation methods. Additionally, other important limitations of this work are: the absence of objective evaluation methods (such as rhinomanometry and acoustic rhinometry), the fact that clinical tests for allergic rhinitis have not been performed, and the absence of a cost assessment for applying the proposed method in clinical evaluations.

## Conclusion

In this study, the NOSE scale was used, in the sixth postoperative month, to evaluate patients who had been treated with either inferior turbinate radiofrequency or lateralization combined with septoplasty. No significant difference was observed between the RF group and the LAT group in terms of relieving the symptoms. Since the two treatment methods do not differ in terms of nasal obstruction symptoms, the authors believe that the treatment method should be chosen at the discretion of the patient and surgeon(s). However, while surgeons choose between these two methods, it should be noted that the radiofrequency adds an extra cost to the procedure and does not provide a significant difference in improvement of the symptoms scores.

## Compliance with ethical standards

The study was performed in accordance with Decleration of Helsinki. Written informed consent has been obtained from all participants.

## Conflicts of interest

The authors declare no conflicts of interest.
